# Humanization of nursing care: a systematic review

**DOI:** 10.3389/fmed.2024.1446701

**Published:** 2024-09-26

**Authors:** Ángeles Reyes-Téllez, Alberto González-García, Adelina Martín-Salvador, María Gázquez-López, Encarnación Martínez-García, Inmaculada García-García

**Affiliations:** ^1^Miguel Servet University Hospital, Zaragoza, Spain; ^2^Department of Nursing, Faculty of Health Sciences, University of Granada, Granada, Spain; ^3^Department of Nursing, Faculty of Health Sciences, University of Granada, Ceuta, Spain; ^4^Virgen de las Nieves University Hospital, Andalusian Health Service, Granada, Spain

**Keywords:** humanization of assistance, nursing care, nurse–patient relations, systematic review, working conditions

## Abstract

Advances in healthcare in recent years have resulted in the automation and standardization of healthcare. Consequently, care has become dehumanized. There is a lack of trust from patients toward the health care system, as well as feelings of stress, exhaustion, and fatigue among professionals. The aim of this article is to describe the humanization actions in nursing care, as well as the barriers and facilitating strategies to carry them out. A systematic review of the scientific literature has been carried out following the recommendations of the PRISMA declaration. The search was carried out in the WOS, SCOPUS, CINAHL Complete, MEDLINE (via PubMed), EMBASE and Cochrane Library databases. The keywords used were “humanization of assistance,” “nursing care,” and “nurse–patient relations,” restricting to original studies in English or Spanish, from 2018 to 2022. One author performed the search, selection, and screening of records. Two authors were involved in data extraction, and a third author decided in case of conflict. The systematic review was guided by ethical conduct that respects authorship and reference sources. Of the 744 articles initially identified, 27 were included in this review. Methodological quality was assessed following the STROBE statement or the CASPe and MMAT tools. The main barriers were found to be the lack of training of nurses and their working conditions, as well as the unwillingness of the institutions. Facilitating strategies consisted of solving implementation problems, promoting communication to strengthen nurse–patient relationships and accompaniment by family members. The main interventions are related to the physical environment and have been studied in obstetrics and pediatrics services. Barriers related to training, work situation and lack of institutional involvement are complemented with facilitating strategies that aim to implement the humanized model at a general level.

## Introduction

Advances in healthcare in recent years have led to increased quality, safety and efficiency in health care (1). These results have been achieved through the promotion of specialization of professionals and the development of technologies for the prevention, diagnosis, treatment and rehabilitation of diseases (1, 2). However, technification has also meant automation and standardization of care and fragmentation of work (1). These elements often lead to a decrease in the time spent on direct care. This has led to dehumanization and depersonalization of care, resulting in a lack of trust from patients toward the health care system and feelings of stress, burnout, and fatigue among professionals (1).

Although this conflict persists today, it began to be studied in the last century. The American Rogers and colleagues (3), between 1938 and 1963, researched client-centered care. The term patient-centered medicine was introduced by Balint et al. in 1970 (4), which was to provide care that was not limited exclusively to the management of signs and symptoms (1, 5). Rogers himself qualified his model as client-centered or person-centered care (1, 3), which implied a more holistic perspective where symptoms and illnesses are treated in the context of each person’s unique experiences. Kitwood and Bredin (6) developed a model in 1992, which highlighted the importance of not only considering the physical state of the person in understanding their behavior. They considered equally significant their biography, personality traits and environmental peculiarities.

Subsequently, the concept of Humanization of Care was introduced, based on the model of Margaret Jean Watson (7), an American nurse considered a reference in the defense and application of the Humanization of Care. In 1979 she published her first book, in which she set out her theory, and in 1999, she structured it in the 10 Caring Factors for the Caritas Process or Process of Caring (8). Her theory belongs to the School of Caring, which defends the possibility of improving care by promoting the dimensions of spirituality and culture (9). This new proposal encompasses care for the person, the elements involved in the process and the interactions between them (1).

Today, Bermejo (10) asserts that respect for the human being is more assiduously guaranteed. Different countries have developed policies to recognize humanized care. Nurses are involved in direct care, management, technological manipulation, etc. They may suffer from work overload, so that humanization is neglected (11). This implies dissatisfaction of nurses, but also of patients, who perceive the need for empathetic care (12).

Other review studies have been conducted on the humanization of care and the role of nursing professionals in making it possible. Some focus exclusively on nurses and how their performance influences the humanization of care (13–16), and others include the multidisciplinary healthcare team or institutions (1, 17–19). Generally, they agree on the need for the promotion of education in nursing professionals, provided during university studies, as well as in postgraduate studies (17, 18).

A systematic review published in 2015, in which the participation of nurses as health educators was studied placed the emphasis on communication with the patient and the creation of an interpersonal relationship. It was concluded that it was an essential part of humanization, considering it a decisive factor for better patient recovery, integration with the team and with the family itself (13). Two other more current reviews coincided with this statement. The first was published in 2020 and considered communication as a mediating instrument for the humanization of care and the establishment of a relationship of trust (15). The second one was published in 2021 and aimed to find scientific evidence on humanized care from the perspective of nurses and in hospitalized patients. It was considered an urgent need for health institutions to be able to guarantee accessibility to humanized health services, as they are linked to patient and family satisfaction (16). The importance of communication between health professionals and family members to humanize hospitalization has also been highlighted (11). Finally, a review study published in 2018 (19) showed that the care environment was as important as the humanization knowledge of professionals. The environment is seen as a tool that makes it possible to provide quality care.

Therefore, although there are reviews on similar topics, it is considered relevant to carry out an update, at a global level, of the experiences to date, in order to generate reflection on the existing advances and gaps in the humanization of care. The systematic reviews that explore the interventions used to implement humanization are scarce. Moreover, there are no systematic reviews that report on barriers and facilitating strategies for the implementation of humanization in a structured way. Existing interventions in the literature reviewed are disparate. For this reason, it has been considered pertinent to carry out a systematic review to compile the published evidence on the characteristics of these strategies. The main objective is to know the advances in humanization that have been made in nursing care, at a global level, in the last 5 years. The following secondary objectives are planned: to describe the interventions used to implement humanization, to find out what the barriers are and, in contrast, to list the facilitating strategies for its implementation.

## Methods

A systematic review of the scientific literature was conducted following the recommendations of the PRISMA 2020 (20) statement, designed primarily to conduct systematic reviews of studies evaluating the effects of health interventions.

### Research question and design

The research question was stated following the PICO (21) structure: P - patients perceiving nursing care as well as nurses providing nursing care; I - interventions to provide humanized care; C - comparison of different interventions implemented to provide humanized care; O - the presentation of humanization in nursing care.

### Search strategy

The WOS, SCOPUS, CINAHL Complete, MEDLINE (via PubMed), EMBASE and Cochrane Library databases were searched for evidence that met the research objectives. The search for articles was restricted from 1st January 2018 to 31st December 2022. Only articles in English and Spanish were accepted. The search was conducted between February and March 2023. The keywords used were “humanization of assistance,” “nursing care,” and “nurse–patient relations,” following the terms DeCS (Descriptors in Health Sciences) for the formulation of search equations in Spanish, and the MeSH (Medical Subject Headings) thesaurus to extrapolate the search to other languages. The three keywords were combined using the Boolean operator AND. The following search strategies were used in all the databases consulted: Humanization of Assistance (AND) Nursing Care, Humanization of Assistance (AND) Nurse–patient relations. WOS, SCOPUS and Cochrane Library proposed default filters related to topic, title, abstract or keywords. Specifically, WOS proposed default filters related to topic (title, abstract and indexing); and SCOPUS and Cochrane Library applied default filters related to title, abstract and key words. These filters were accepted in our searches, except in Cochrane Library, where the search was only filtered by abstract. Similarly, researchers applied equivalent restrictions in the other three databases consulted: EMBASE was searched by title, abstract and keywords; MEDLINE (via PubMed) was searched by title and abstract; and CINAHL Complete was searched by abstract.

### Selection criteria

We included studies on activities to humanize nursing care, published in English or Spanish, from 2018 to 2022 (both included), both quantitative and qualitative. We excluded studies that addressed interventions to humanize care that did not involve nursing staff, as well as those that focused on interventions delivered by nurses that were not related to the humanization of care. Records with limited possibility to provide relevant information or low methodological quality were excluded. We considered low quality articles to be those with less than 50% of the items in the STROBE (22) and CASPe (23) tools, or a negative result in the control questions of the MMAT (24) questionnaire.

### Data extraction process

One author of this review was responsible for searching, selecting, and screening the records (ART). Two authors were involved in data extraction (AGG, IGG), with a third author deciding in case of conflict between them (AMS).

### Quality analysis

The assessment of the methodological quality of the articles included in the review was carried out following the standards and criteria of the STROBE (22) statement for descriptive cross-sectional studies, with the CASPe (23) tool for those with a qualitative design and with the MMAT (24) tool for mixed methods studies. A consensus was reached among the authors to relate the total score obtained to the quality of each article. Articles that met less than 50% of the items were considered low quality, between 50 and 75% moderate quality and more than 75% high quality.

### Risk of bias analysis

The analysis was carried out by peer review by two different authors (MGL, EMG). An intensive reading and detailed analysis of the information described in the studies was carried out with the help of tools to determine the risk of bias (RoB). AHRQ tool (25) was used for the evaluation of cross-sectional studies. A consensus was reached among the authors to relate the total score obtained to the risk of bias of each article. A score from 0 to 4 indicates a high RoB, from 5 to 7 indicates a moderate RoB and from 8 to 11 indicates a low RoB. The JBI checklist (26) was used for qualitative studies. A score from 0 to 4 indicates a high RoB, 5–7 indicates a moderate RoB and 8 to 10 indicates a low RoB. Finally, MMAT (24) tool was used for mixed methods studies. A score less than 50% indicates a high RoB, 50–70% indicates a moderate RoB and 80–100% indicates a low RoB.

### Ethics approval and informed consent statement

This article does not contain any studies with human or animal participants and informed consent is not required.

## Results

**Figure 1:**
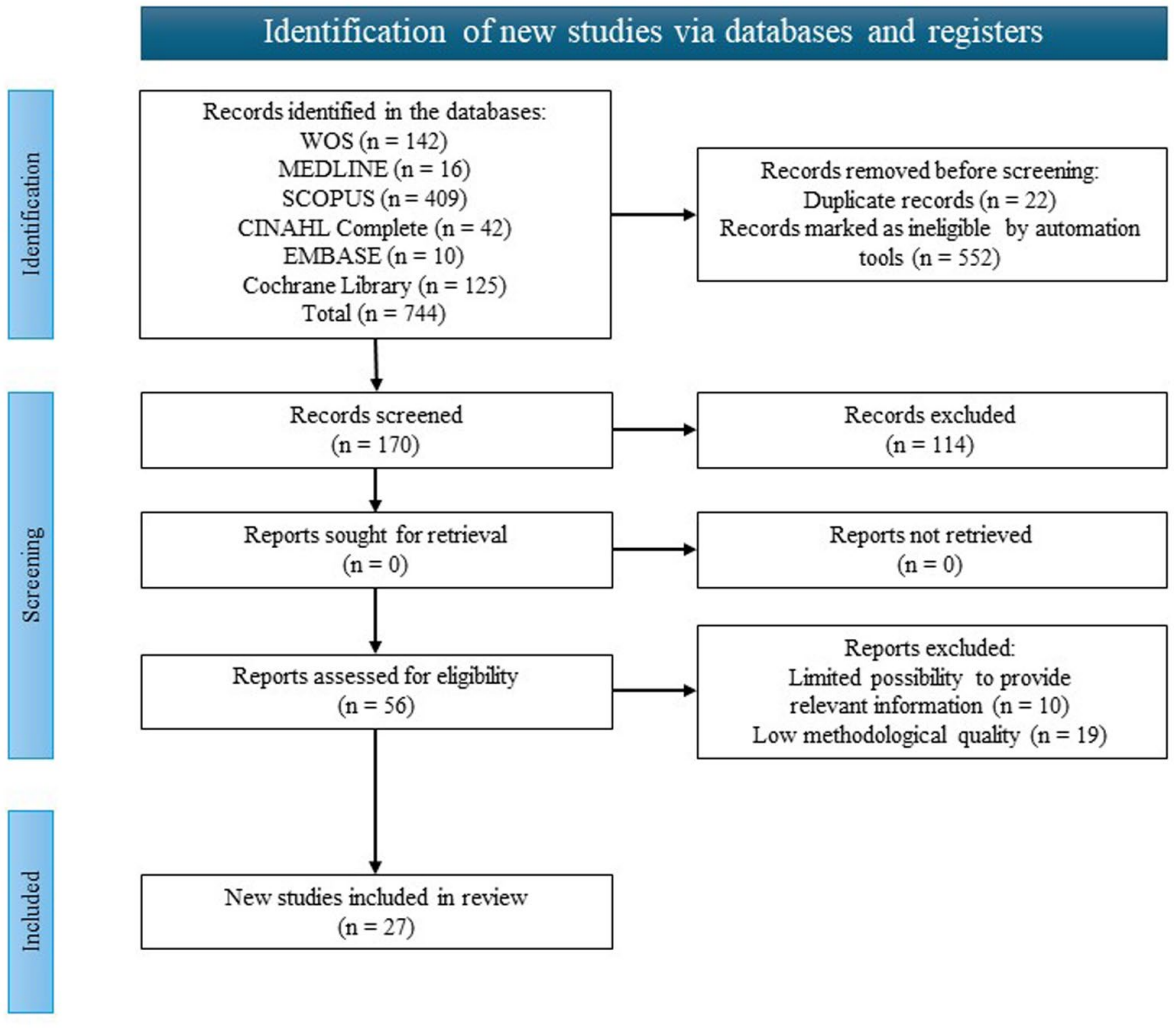
Flow chart.

The initial search of WOS, MEDLINE (via PubMed), SCOPUS, CINAHL Complete, EMBASE and Cochrane Library databases resulted in a total of 744 records (WOS *n* = 142, MEDLINE (via PubMed) *n* = 16, SCOPUS *n* = 409, CINAHL Complete *n* = 42, EMBASE *n* = 10, Cochrane Library *n* = 125). Filtering by inclusion and exclusion criteria resulted in 192. Removing duplicate articles resulted in 170 articles. Articles were screened based on titles and abstracts, resulting in 56 articles from WOS, SCOPUS, and CINAHL Complete. Finally, a complete and exhaustive reading of all articles was carried out to select 27 articles. Two authors participated in the final selection, and there was no disagreement between them regarding the inclusion of studies in this systematic review. No publications were retrieved. They were presented according to the recommendations of the PRISMA 2020 statement (Figure 1). With the research question as a guide, information was extracted from these articles to analyze and interpret them.

The 27 selected articles were carefully reviewed and ordered. An analysis was made of the most relevant data, as well as of their study process: methodology, sample, results, conclusions obtained, score achieved and correspondence with their methodological quality and risk of bias (Table 1). The articles were published in six countries: Brazil (27–42), Spain (43–47), Chile (48, 49), Portugal (50), Canada (51), Mexico (52), and Iran (53). It includes 15 qualitative studies (27, 28, 30–33, 35, 37, 38, 40, 41, 44–46, 51, 53), 10 quantitative (29, 34, 36, 39, 42, 43, 47–49, 52) and two mixed (30, 50) which were critically analyzed for inclusion in the study. The topics addressed by the studies are lack of nurse training (29, 31, 37, 42, 50), working conditions (27, 37, 42, 51, 52), institutional unwillingness (27, 29, 37, 38, 42, 51, 52), strategies for solving implementation problems (27–30, 36–38, 41–44, 46, 49, 51–53), promoting communication to strengthen nurse–patient relationships and encouraging patient autonomy (27, 30, 33, 34, 38, 40, 41, 44, 46, 48, 49, 52, 53) and family member accompaniment (28, 29, 31, 34–36, 39).

**Table 1:** Summary of the analysis of the articles included in the review.

Authors (year)	Country	Methodology	Sample	Discussion and conclusions	Quality Score	Risk of bias
Ferreira et al. (27)	Brazil	Qualitative, semi-structured interview, non-participant observation	5 nurses 5 nursing technicians	Possibility of humanizing childbirth with good practices, which do not depend on technology or large investments. Difficult to implement due to shortage of skilled professionals, problems with institutional routine and lack of an obstetric center	CASPe 7 Moderate	JBI 5 Moderate
Silva Junior et al. (28)	Brazil	Qualitative, semi-structured interview	7 nurses	Importance of the environment that favors humanization. Thanks to music therapy, signs of stress, depression and irritability of the patient decreased. It also benefited the professionals	CASPe 7 Moderate	JBI 7 Moderate
Melo et al. (50)	Portugal	Qualitative-quantitative, mixed questionnaire, quasi-experimental study	64 nursing students	Nursing students can benefit from the development of relational skills through training, thanks to the application of the MCH tool	MMAT 3 Moderate	MMAT 3 Moderate
Santos et al. (29)	Brazil	Quantitative, cross-sectional, exploratory and descriptive	22 nurses 61 patients’ relatives	Nurses recognized that their knowledge of spiritual care was insufficient. The lack of an integrated plan made it difficult to incorporate humanized care. Spiritual care encouraged the inclusion of the family member actively in the process	STROBE 17 High	AHRQ 6 Moderate
Soares et al. (30)	Brazil	Qualitative-quantitative, semi-structured interview, descriptive	1 head nurse 14 auxiliary nurses 2 routine nurses 1 nursing resident 28 nursing technicians	It is important to build a trusting relationship with the pediatric patient, to play, to speak kindly, to encourage explanations and to include the pediatric patient in decision making. The experience of the professional and the environment to provide comfort are very important	MMAT 2 Low	MMAT 2 High
López-Tarrida et al. (43)	Spain	Quantitative, descriptive, cross-sectional	99 nurses 90 TCAE 113 doctors	It is considered of great interest that professionals are trained in the detection of needs in the spiritual/religious dimension	STROBE 16 Moderate	AHRQ 5 Moderate
Soares et al. (31)	Brazil	Qualitative, semi-structured interview, descriptive, intervention	24 family members	The neonatal welcome protocol is an important tool for the humanization of care. It mitigates feelings of uncertainty at the parents’ first visit and encourages their early involvement	CASPe 7 Moderate	JBI 5 Moderate
Barroso et al. (32)	Brazil	Qualitative, unstructured interview	7 patients	The therapeutic toy contributes to the process of interaction between the child and the nurse. It improves coping with the need for venipuncture, reduces anxiety and stress, and promotes cooperation and adherence to treatment	CASPe 6 Moderate	JBI 6 Moderate
Rocha et al. (33)	Brazil	Qualitative, semi-structured interview	24 patients	The midwifery consultation promotes positive results in the prevention of cervical cancer. It is characterized by understanding of the patient, which promotes her autonomy in decision-making and management of her health	CASPe 7 Moderate	JBI 6 Moderate
Coelho et al. (34)	Brazil	Quantitative, descriptive, cross-sectional	100 family members	The most important needs of relatives of patients admitted to the coronary ICU were related to safety and information. Family involvement improves decision making and communication with the team	STROBE 16 Moderate	AHRQ 6 Moderate
Souza et al. (35)	Brazil	Qualitative, descriptive, exploratory, semi-structured interview	21 family members	Welcoming protocols should be promoted in hospitals that encourage the role of the pregnant woman and her companion, as well as expanding the information that the mother receives during the gestation process. Lack of preparation generates insecurity, doubt and anxiety	CASPe 7 Moderate	JBI 6 Moderate
Navarrete-Correa et al. (48)	Chile	Quantitative, descriptive, cross-sectional analytical	51 patients	It was felt that in the oncology department there was a need for nurses capable of providing both technical and emotional support (especially willingness to communicate). More than half of the patients felt that they always perceived humanized care, which increased the quality of the person’s existence	STROBE 17 High	AHRQ 5 Moderate
Guillaumie et al. (51)	Canada	Qualitative, descriptive, exploratory	10 registered nurses 7 auxiliary nurses	Most of the nurses reported negative feelings resulting from dehumanization. More experienced nurses tended to be more comfortable providing care. Institutional policies to promote and integrate humanized care and to ensure adequate staffing are recommended	CASPe 9 High	JBI 7 Moderate
Reynaga-Ornelas et al. (52)	Mexico	Quantitative, descriptive, cross-sectional	103 patients	Need to train nurses in humanized care and implement communication as a tool for humanization. This care is affected by physical infrastructure and working conditions. One strategy is to adjust the nurse–patient ratio	STROBE 18 High	AHRQ 5 Moderate
Esteban-Sepúlveda et al. (44)	Spain	Phenomenological qualitative, semi-structured interview	15 patients	Humanized care fosters the nurse–patient relationship, increases the information women receive, which results in women wanting to be actively involved. It is important to promote institutional strategies to empower professionals and to carry out continuous evaluation of the process	CASPe 9 High	JBI 9 Low
Paiva-Nóbrega et al. (36)	Brazil	Quantitative, observational, cross-sectional	55 patients	There has been progress in the quality of delivery care, but there is a need to extend information to women and to encourage professionals to adhere to good obstetric practices	STROBE 16 Moderate	AHRQ 7 Moderate
Maia et al. (37)	Brazil	Qualitative, focus group	55 nurses	Therapeutic play provides effective nursing care for pediatric patients, reducing anxiety, fear, and pain. It should be implemented in care plans and motivate professionals	CASPe 8 High	JBI 7 Moderate
Zamaniniya et al. (53)	Iran	Qualitative, semi-structured interview	16 nurses	Thanks to humanized care, patients had a better opinion of nurses, increased their satisfaction and comfort. It is recommended to integrate courses on humanization in the nursing curriculum	CASPe 8 High	JBI 7 Moderate
Hernández-Garre et al. (45)	Spain	Qualitative, participant observation, semi-structured interview	20 patients	Transition processes are taking place in the clinical networks of institutional delivery. These are hybrid clinical networks that integrate humanization interventions but are based on an interventionist logic and medicalization of childbirth	CASPe 9 High	JBI 7 Moderate
Silva et al. (38)	Brazil	Qualitative, descriptive approach	20 nurses (postgraduate students)	The education of nurses and the promotion of information for mothers has led to a decrease in cases of obstetric violence. Strengthening the model of care implies promoting the humanization training of professionals	CASPe 7 Moderate	JBI 7 Moderate
Alvares et al. (39)	Brazil	Quantitative, cross-sectional	104 patients	The results show quality nursing care and increased visibility of their work. Practices for the humanization of care promote maternal well-being	STROBE 16 Moderate	AHRQ 5 Moderate
Anguita et al. (46)	Spain	Qualitative, semi-structured interview	11 nurses	The patient must play a more active role in decision-making processes about their health. It is important that institutions offer training programs in humanized care. There is a move toward more holistic models of care	CASPe 10 High	JBI 8 Low
Gros-Polo et al. (47)	Spain	Quantitative, descriptive, cross-sectional	34 nurses 42 patients	Patients place more value on care than nurses do. There may be a lack of motivation on the part of professionals to progress in care, and a lack of job rewards	STROBE 17 High	AHRQ 6 Moderate
Torres et al. (40)	Brazil	Qualitative, descriptive, semi-structured interview	34 patients	Emphasizes the importance of nurses’ skills as a central element of care. By providing information to patients, shared decision making is encouraged, and greater patient autonomy is achieved	CASPe 6 Moderate	JBI 6 Moderate
Colaço et al. (41)	Brazil	Qualitative, exploratory, descriptive, semi-structured interview	16 nurses	It is important for the nurse to establish a bond with the patient, to forge a relationship of trust and to inform the patient openly, promoting patient autonomy. Emotional preparation is needed for the anxiety and stress generated by the disclosure of bad news	CASPe 8 High	JBI 6 Moderate
Monje et al. (49)	Chile	Quantitative, cross-sectional, correlational	171 patients	Nurse–patient bonding is critical. This allows positive feedback for the professionals. Nursing must be underpinned by humanized, timely and quality care	STROBE 14 Moderate	AHRQ 6 Moderate
Cordeiro et al. (42)	Brazil	Quantitative, field-based, descriptive and exploratory	30 nurses	Difficulties were encountered in providing care. Despite these limits, nurses are carrying out humanization actions in childbirth and providing quality care	STROBE 14 Moderate	AHRQ 4 High

Three different tools with different assessment criteria were used to analyze the quality of the studies. The CASPe (23) tool was used for the analysis of qualitative studies. It is a tool with 10 items. Seven studies showed high methodological quality, obtaining scores of eight (37, 51, 53) nine (44, 45, 51) and ten (46). The majority obtained moderate quality with scores of six (33, 40) and seven (27, 28, 31, 33, 35, 38). Quantitative studies were assessed following the STROBE (22) statement. Four of them showed high methodological quality, meeting seventeen (29, 47, 48) or eighteen (52) criteria. The others were considered to be of moderate quality as they met 16 criteria (34, 36, 39, 43), except for Monje et al. (49) and Cordeiro et al. (42), which only met 14. The quality analysis of the mixed studies was performed using the MMAT (24) tool. In the control questions, a positive result was achieved in both, so we continued with the questions according to the study design category. In the first (50), some shortcomings were noted in the rationalization of the rationale for using a mixed approach. It is also felt that the integration of qualitative and quantitative data could be more unified. Its methodological quality is considered to be moderate, meeting 60% of the items. In the second (30), shortcomings have been observed in the explanation of the relevance of the use of mixed methods to answer the research question. The objective data are not presented in an integrated way. It is considered to be of low methodological quality, due to 40% of the criteria. As mentioned before, the risk of bias (RoB) of quantitative studies was measured using the AHRQ (25) tool for cross-sectional studies. All quantitative studies showed a moderate risk of bias (29, 34, 36, 39, 43, 47–49, 52) except for the article by Cordeiro et al. (42), which reported a high risk of bias. The risk of bias of qualitative studies was determined using the JBI (26) checklist. Two studies had a low risk of bias (44, 46), while all others showed a moderate risk of bias (27, 28, 31–33, 35, 37, 38, 40, 41, 45, 51, 53). The mixed methods studies were measured with the MMAT (24). As explained in the quality section, both showed positive results on the control questions. The first article (50) had a moderate risk of bias, whereas the second article (30) a high risk of bias. Although there is a disparity in the quality and risk of bias of the studies, it was decided to include them in this review because they provided relevant information to answer the stated objectives.

### Interventions used to implement humanized care

The studies included in this review show an intention to change toward more humanized care. In some, a few variations in care are made, in others, major modifications of protocols. Often the actions are directed toward a specific type of care.

Among the most frequent activities are those related to the physical care environment. Examples are the adequacy of light, noise, or temperature. The nursing staff themselves can also provide a supportive environment by grouping all interventions together at one time, making postural changes, using cushions to improve posture, or providing adequate body hydration (30).

Humanization actions in the obstetric setting are among the most studied. The importance of non-invasive techniques (39), non-medicalization of labor and reduction of unnecessary interventions are emphasized (27, 36). Pain relief can be provided through non-pharmacological procedures (early skin-to-skin contact or positions for pain relief), individualization of labor through dialog with the laboring woman, free choice of companion or effective emotional support. Importance is also given to physical measures, such as restricting certain procedures (episiotomy or speeding up labor), favoring the choice of birth position (encouraging upright positions), using rubber balls, massaging, late umbilical cord clamping, promoting breastfeeding, providing a low-stimulation environment, taking warm baths, walking, or promoting music therapy. Similarly, the use of non-invasive tools, such as the partogram, is encouraged for accurate monitoring of labor (27, 36, 42, 45).

Similarly, actions to humanize the care of pediatric patients are explored. They need affection and reassurance as part of care, which can be more humanized if nurses include playing, talking with kindness and providing both information and explanation of procedures (30). A good example is the use of dolls in therapeutic play as mediators to facilitate care. It promotes adherence to treatment and children’s cooperation because they understand the process better. This reduces the anxiety and stress they feel, and promotes resilience (32, 37). Distraction strategies such as videos, books, toys, and songs are also used while the techniques are being carried out. With these actions, the hospital routine is transformed into a more familiar experience, so that they have fun in a context of normality (37).

### Barriers to the implementation of humanized care

Despite the activities undertaken to achieve the humanization of care, many of the articles analyzed in this review report difficulties in implementation. Barriers related to nursing staff knowledge are noted (29, 31, 37, 42, 50). The importance of such knowledge was described in research comparing the relational skills of students who had received training in humanized care and those who had not, which concluded that there was a need to implement methodological teaching that fosters the skills to provide complex humanized care (50). This problem was also noted in a study on therapeutic play (37). The training deficit is a drawback that continues throughout specialization and professional practice. In several studies on humanization in delivery care, professionals were poorly prepared, had insufficient knowledge of procedures, or were demotivated (29, 37, 42). This motivation influenced the quality of care, as the willingness to perform care is as important as training (31). In addition, the work of the nurse is often undervalued (31). In one study it was found that patients and nurses perceived care differently, with patients rating the care higher (47).

Similarly, the work situation of nurses resulted in difficulties in providing humanized care. Nurses reported having few professionals and, in addition, staff turnover was low. Therefore, patient care time was more limited (27, 37, 42, 51, 52). In a study on nurse empowerment, nurses themselves recommended increasing staff recruitment, considering lack of time a problem, in addition to inadequate remuneration (51). In another study on spiritual care for critically ill patients, nurses reported lack of time as a barrier (29). Sometimes this lack of time was not due to the need for more staff, but to the amount of administrative tasks that nurses had to perform, which reduced direct care and nurses reported feeling ‘burnt out’ (27, 37, 51). The emotional health of workers was also found to be influenced by negative feelings resulting from the dehumanization of care, such as frustration, anger or diminished empowerment (51). This dehumanization was reported by patients, who, in a study on the evaluation of nurses’ humane care, rated nursing techniques higher and considered humanized care less common (52).

Other aspects described in the studies were problems related to the unwillingness of institutions to implement humanized care. Thus, some studies point out that management support is needed to solve the problem of the physical structure, which was considered inconsistent with the humanization policy, as well as to address the lack of material resources and the need for training of nurses. At other times, it was the lack of an integrated plan for daily care that affected the possibility of incorporating humanized care (27, 29, 37, 42, 51, 53). Practical examples included prioritizing other activities over the humanization of care during labor, not providing the mother with the necessary privacy or not allowing her to choose her companion (27). In a study on the same topic, some women reported a lack of clarity and completeness in the guidelines provided to them (38), although in another study they rated the availability of resources during labor as positive (44).

### Facilitating strategies for the implementation of humanized care

A large proportion of these were aimed at addressing the lack of training of nurses. Among the possible solutions, several studies proposed that there should be a qualified humanized care professional dedicated exclusively to this task (27, 29), although a majority of studies advocated encouraging the training of all nurses (36, 38, 43, 44, 52).

It has been observed that more experienced nurses tended to be more comfortable providing humanized care, as they were more familiar with the technical aspects and had experience with a variety of situations (29, 30, 51). It is proposed that humanization training should begin in undergraduate studies, integrating courses into the nursing curriculum (38, 53). The importance of continuing with postgraduate training has been highlighted (38, 42). Among the contents of this training, communication with the patient (41, 46) and the professionals’ ability to cope have been mentioned (46). Actions that allow positive feedback on the activities that are carried out are contemplated (28, 37, 49).

An essential skill of the nurse is the ability to communicate, considered the key tool to achieve the humanization of care (48, 52, 53). This was observed in a study on the needs of family members of critically ill patients in which communication and support received, even in situations of little clinical progress, were predictors of satisfaction (34). The more information nurses provide, the more confident the person being cared for feels, increasing their trust in the professional (44). It favors the creation of a bond between nurse and patient, which helps to minimize their anxiety, fear, insecurity and doubts (38, 41). Likewise, patients perceive that professionals invest time in them (49).

Adequate communication allows people to have more autonomy in decision-making and to participate in managing their own health, adopting a more active role (30, 33, 46). The patient must have maximum information and be involved in the process (40). One tool to promote this autonomy is the implementation of continuing education strategies (41). Several examples are noted in obstetric studies describing the importance of promoting maternal empowerment (27, 44). The provision of information should begin in prenatal care and continue through the postpartum period, in order to restore the mother’s agency (27, 35, 36, 39). There is also a need to encourage the autonomy of the companion, as this has been found to reduce the practice of intervention (35). Similarly, there are benefits to including a companion or other family members in care (36, 39). This has been shown in studies on the individual distress of patients with COVID-19 hospitalized in ICU, which was worse when unaccompanied by their relatives (28). Those relatives who actively cooperated in the care process felt empowered, had better communication with the professional team and felt less fear and hesitation at discharge (29, 31, 34).

The involvement of institutions is considered an essential facilitator for the promotion of humanized care. The importance of providing humanized spaces and training professionals is highlighted. Even so, different studies have shown the need for the administration to invest more resources in these aspects and to improve the management of existing infrastructures (28, 37, 46). The need for greater recognition is also projected, due to the fact that humanized care is sometimes not reflected, although it takes time and results in benefits for patients (47). The need to implement all these actions at a general level is projected, so that these processes can be carried out, valued and disseminated at the hospital level. In this way, the humanization of care would be more homogeneous in all services and would not depend on the individual changes made in each one (31, 33, 35, 37, 41, 44, 51). A summary of the different barriers and facilitators identified are presented in Table 2.

**Table 2:** Barriers and strategies identified.

Barriers to the implementation of humanized care	Facilitating strategies for the implementation of humanized care
Lack of nursing training (in undergraduate studies and specialization and professional practice)	Encouraging the training of all nurses
Undervaluation of nursing practice	Using communication as a tool for humanization: informing the patient, encouraging the patient autonomy and creating a bond with the patient
Inadequate working conditions: low staff turnover, patient care time limited, inadequate remuneration	No strategy to overcome the barriers to provide humanization has been found
Negative nurse feelings: “burn out,” frustration, demotivation, anger or diminished empowerment	Empowering the patient and include family members in care
Unwillingness of institutions to implement humanized care: problem of the physical structure and in the humanization activities	Greater involvement of institutions in providing the tools to humanize care

## Discussion

The aim of this systematic review was to learn about the advances in humanization that have been made in nursing care, at a global level, in the last 5 years. Although there were systematic reviews already published on this same subject, the secondary objectives proposed here differ from those published previously, so the knowledge provided is considered of great relevance. This systematic review has highlighted the importance of humanizing care and demonstrates the efforts of health services and professionals to achieve this. Most of the studies highlight the importance of institutions in promoting change.

### Most relevant interventions used to implement humanized care related to environment, healthcare providers and the most studied care units

The importance of the interventions used to humanize care, both in terms of changes in the environment and in the way in which they are carried out, is discussed. The importance of such interventions is reiterated in the literature. Most research does not explicitly emphasize their characteristics or lumps them together with change strategies.

Despite their importance, only one study has been included in this systematic review focusing on the influence of the physical environment in which care is perceived (30). Even so, the results obtained coincide with the contributions to promoting the well-being of pediatric patients listed in a review conducted in 2014 (19) that aimed to analyze strategies to humanize the care of hospitalized children. Leisure practices such as toys, music and reading were found to minimize the stress of hospitalization and produce a calming effect. Children were encouraged to participate in the decoration of healthcare facilities in order to humanize the environment. Subsequent research has followed the same line, as is the case of the research study included in the present review (30). Others placed greater importance on the actions of healthcare staff, as is the case of research on the perception of the companion of the child in hospital in a pediatric intensive care unit, published in 2017. A greater perception of humanized care was associated with healthcare workers being actively involved in the care (54).

Most of the studies included in this review have been conducted mainly in obstetrics (26, 33, 36, 39, 42, 45) or pediatric services (30, 32, 37), detailing interventions focused on fostering more humanized care. One study on gynecological reception stands out, in which it was argued that dialog facilitated the process of humanization (33). This consists of receiving the person with an attitude of closeness in which dialog is essential. This was agreed in a review of 2020 (11) which aimed to understand the behavior of nurses in implementing hospital humanization and another review on the challenges of nursing for universal health coverage, published in 2016 (14). The latter revealed that a greater welcome was achieved in those units that had been carrying out this action for longer, in which patient access to the unit was encouraged and in which multidisciplinary team meetings were held to evaluate the services.

### Lack of nursing knowledge and training, poor working conditions and institutional obstacles as significant barriers to humanizing care

Many studies that form part of this review agree that the lack of training of nurses is one of the main barriers to being able to provide humanized care (29, 31, 37, 42, 50). They refer to the existence of this problem from undergraduate training, although they do not go into too much depth. However, in an integrative review of the literature published in 2020 (15), they reflect on how unprepared students feel. They consider that there is a need to supplement the knowledge provided theoretically with occasional seminars and lectures. These findings are in line with those of an earlier review from 2012 (18). It states that including a humanization strand in an isolated course is insufficient to achieve significant changes. Along the same lines are the results of a 2018 research study (17). It confirms the fact that there is no university policy to raise students’ awareness of humanization. Additionally, postgraduate training does not address this training deficit. As a result, nurses feel underprepared, which leads to demotivation and a decrease in the quality of care (31). The results of a 2016 systematic review (14), conducted with the aim of identifying nursing objectives for universal health coverage, also coincide with the above. It reiterates the need to train nurses and all professionals in humanization. A 2021 systematic review, which investigated nurses “and patients” perspectives on humanized care (16), concluded that care recipients perceived limitations in care due to lack of social skills, selflessness and low compassion in the face of suffering. These competencies of professionals were assessed to a greater extent in studies that investigated the patients’ point of view (1).

The articles agree that a major barrier to care is the working conditions in which nurses provide care. Limited care time results in dehumanization, which is related to insufficient staffing and management (27, 37, 42, 51, 52). Different research corroborates the findings of the present systematic review on the influence of nurses’ working conditions on humanization (1, 11, 15, 17, 19). Understaffing is related to increased demand for care, which results in work overload, little time to provide humanized care, intense work routine and increased bureaucratic demands. This is associated with increased stress and job dissatisfaction (1, 11). All of this influence patients’ views of nurses, who consider humanized care less common than the performance of techniques (52).

The importance of management involvement is underlined. The training provided to practicing professionals is considered insufficient (29, 37, 42, 51, 52). Other publications also relate this aspect to involvement in the well-being of professionals (e.g., offering psychological support), although there is currently a gap in this area (1, 19). The low willingness observed on the part of the institutions in this study coincides with that obtained in other reviews. As in the present systematic review, an integrative literature review from 2022 (55) found barriers related to the lack of institutional support. This review investigated the role of nurses with patients who are experiencing maltreatment. Nurses felt that it was important to create opportunities to address barriers related to work overload, lack of professional preparation to identify cases and non-recognition of violence as a health problem. This led to feelings of helplessness and insecurity among nurses.

Infrastructure-related barriers were closely linked to difficulty in accessing services, which negatively affected patients’ confidence and influenced the possibility of creating a therapeutic bond with professionals (1, 13–15, 19). A Brazilian systematic review published in 2015 (13) evaluated the involvement of the nurse as a health educator and primary care provider. It reflected institutional barriers influencing humanization, which were consistent with the results of this study, including physical structure, deficiencies in protocols and organizational shortcomings. Other barriers included long waits or delays, both for consultations and examinations, and deficiencies in routines, center rules and equipment (13).

### Promoting nursing knowledge and training, use communication as a humanizing tool, including the family in care and improving institutional participation as facilitators of humanized care

As a solution to the lack of training in humanization observed in most of the articles, its promotion is proposed as a facilitating strategy. The experience of nurses was related to a more favorable situation for providing humanized care (29, 30, 51), so one strategy was to provide nurses with knowledge to help them in dealing with different situations. In the literature consulted, it has been observed that many of the interventions to promote training are aimed at undergraduate studies. The focus is on learning techniques based on participatory and reflective methods, which encourage student autonomy and teamwork. To achieve this, practical humanization activities and teacher involvement are advocated (14, 15, 18). Of particular note is a 2012 critical review on the teaching of humanization of care in undergraduate programs, in which different Brazilian publications were analyzed (18). It includes teaching strategies such as dramatic games, socio-drama, role-playing, debate, simulations and discussion of films. Emphasis is placed on students being able to reflect on their feelings and limitations (18). Jean Watson stated that those who fail to recognize their own feelings will find it difficult to understand another person’s feelings, so this reflective practice is part of the professionalization of nurses (15). On the other hand, there is a need for continuing postgraduate education of nurses. Institutions have a responsibility to implement the competencies of professionals in humanization, offering specific training in this area, as well as to promote the understanding and participation of professionals and patients (13, 14, 16).

On the other hand, the importance of communication as a fundamental tool for humanization (30, 33, 46) as presented in the results of this study is consistent with a large body of research (1, 11, 13, 15, 16). It was observed that when patients’ preferences and needs were considered, their satisfaction, empowerment, quality of life and improved treatment outcomes were increased (1, 15, 16). The results of this study are consistent with those of an integrative review published in 2022 (55). In this review, the performance of nurses in family health strategies with abused children was investigated. The importance of forming a bond with pediatric patients, in order to obtain details, could break the cycle of violence. All this is directly related to the provision of information to patients, so that they can be involved in the process and their autonomy in decision-making is promoted (30, 33, 40, 41, 46). Specifically in interventions carried out in obstetric services, decision-making during childbirth is related to the empowerment of the mother, in order to promote her protagonism (27, 44). The same conclusions are observed in a systematic review about the nursing perspective of the humanized care of the neonate and family published in 2021 (56). It is argued that professionals should be actively involved in humanized care. In addition, the patient and family should be included in decision making, even being able to discuss the daily care plan and the expected outcomes. As a result, care becomes safer and more efficient.

This systematic review highlights the benefits for patients of including family members or companions in care (28, 29, 31, 34). This information coincides with that published in a review on training guidelines for humanized care, carried out in 2020 (15). It shows that the presence of family members is an element that favors the physical and emotional recovery of the person being cared for. It has been proposed that the family be included in undergraduate programs so that nursing students acquire skills to work with them and value their importance.

It has been mentioned previously that the lack of institutional involvement is a barrier to the implementation of humanized care. Reversing this becomes a facilitating strategy. It is possible to improve the infrastructure to favor humanization and to optimize the use of existing resources for this purpose (28, 37). It is also considered a step forward to offer training programs in humanized care and non-technical competencies (46). Another measure considered necessary is the recognition of the humanization activities carried out by nurses (47). This can be achieved through the promotion of institutional strategies, as well as the implementation of good clinical practices, care plans and welcome protocols. Subsequent assessment of these processes is essential for ongoing evaluation and dissemination of research in the fields of knowledge (31, 33, 35, 37, 41, 44, 51). The literature also stresses the importance of implementing institutional policies governed by quality indicators, which should correspond to international standards of humanization, as well as making research-based changes and applying tools and models to improve care (14, 16).

Finally, it is significant to underline the scarce information that has been found on humanization facilitating strategies related to the work situation of nurses. This review presents strategies to overcome barriers related to the lack of training of nurses or the unwillingness of institutions to implement humanized care. However, there are no strategies that correspond to the barrier of the work situation in which nurses carry out their profession. There is no discussion of possible solutions to the lack of time and staff available to provide care, the inadequate remuneration of nurses or the number of administrative tasks that reduce direct care. However, recognition of the humanization activities they perform is raised (47). There is a big difference between the projection of the future to solve other barriers and the scarcity of this same aspect that has been found in relation to the employment situation of nurses. In contrast, the literature consulted suggests measures such as improving the working conditions of professionals and the relationships between all those involved in the care process (19). To this end, it is important to have a suitable environment that fosters interpersonal relationships and provides welcoming care (1, 19).

## Limitations

This systematic review has some limitations that need to be highlighted. Firstly, the location of the studies that have been analyzed. Most of them have been located in Brazil, which implies that the social and political reality of this country has a greater weight in the vision of humanization provided by this study. Therefore, extrapolation of these results to other geographical areas and socio-cultural environments may be difficult. This limitation may be related to the quantity, quality and impact of the research selected for this review, which does not include gray literature in the search. In this sense, some humanization actions implemented are not subsequently published. Therefore, it would be necessary to complement the information obtained with protocols of each service and with actions included in institutional humanization plans, for example. Secondly, although all the research included in this review studies the humanization work of nursing professionals, some of them also include other professionals such as nursing technicians, auxiliary nursing care technicians or doctors, or nursing students. Nurses perform different tasks and have different levels of competencies depending on the country in which they practice their profession, delegating or taking over delegated actions from other professionals. Thirdly, even though, the studies included in this review have disparate quality criteria. In our systematic review we have included studies that had sufficient quality; therefore, the conclusions of our study do not reflect the individual value of each article. Nonetheless, it is possible that some of the information in these studies was biased or incomplete. Fourth, the search was limited to articles published in English or Spanish, so it is possible that publications in other languages could have met the inclusion criteria and provided relevant information. Finally, it is noted that most of the articles analyzed used a qualitative approach, so the results obtained cannot be extrapolated to the general population.

This review shows the reality of the humanization of care today. The results obtained are not extrapolable to a global level for the reasons mentioned above, although they provide important data on the degree of implementation of humanization. It is considered that in the future it would be pertinent to continue to expand knowledge on this subject. Knowing the perspectives of professionals, patients and relatives provides a richer and deeper vision of the situation, as it encompasses a large part of the factors that influence the care process. It is especially important to know what patients think, since they are the main recipients of care, and it is a way of ensuring that they continue to be protagonists in their vital processes.

## Conclusion

Different interventions have been described to implement humanized care, often targeting a specific type of care. Many of them relate to the environment in which care is provided. Of particular note are those implemented in the field of obstetrics, which are based on dialog and encouragement of patient autonomy, as well as changes in nursing care processes to reduce unnecessary interventions, use less invasive tools and promote physical measures. Interventions in pediatric services that promote information and distraction strategies for painful processes are also highlighted. The barriers encountered have been the lack of training of nursing professionals, the work situation of nursing professionals, with few staff and therefore little time dedicated to humanized care, which generates negative feelings in the professionals, as well as the low willingness of the institutions to change this situation. The facilitating strategies for humanized care are related to the promotion of nurse training, the use of communication as an indispensable tool to achieve patient autonomy in decision-making, the promotion of family accompaniment and greater involvement of the institutions to evolve toward a model of humanized care at a general level.

## Data Availability

The original contributions presented in the study are included in the article/supplementary material, further inquiries can be directed to the corresponding author.
